# Unveiling diabetes onset: Optimized XGBoost with Bayesian optimization for enhanced prediction

**DOI:** 10.1371/journal.pone.0310218

**Published:** 2025-01-24

**Authors:** Muhammad Rizwan Khurshid, Sadaf Manzoor, Touseef Sadiq, Lal Hussain, Mohammed Shahbaz Khan, Ashit Kumar Dutta

**Affiliations:** 1 Department of Statistics, Islamia University College, Peshawar, Khyber Pakhtunkhwa, Pakistan; 2 Centre for Artificial Intelligence Research (CAIR), Department of Information and Communication Technology, University of Agder, Kristiansand, Grimstad, Norway; 3 Department of Computer Science & IT, Neelum Campus, The University of Azad Jammu and Kashmir, Athmuqam, Azad Kashmir, Pakistan; 4 Department of Computer Science & IT, King Abdullah Campus, The University of Azad Jammu and Kashmir, Muzaffarabad, Azad Kashmir, Pakistan; 5 Children’s National Hospital, Washington, DC, United States of America; 6 Department of Computer Science and Information Systems, College of Applied Sciences, AlMaarefa University, Ad Diriyah, Riyadh, Kingdom of Saudi Arabia; Nazarbayev University, KAZAKHSTAN

## Abstract

Diabetes, a chronic condition affecting millions worldwide, necessitates early intervention to prevent severe complications. While accurately predicting diabetes onset or progression remains challenging due to complex and imbalanced datasets, recent advancements in machine learning offer potential solutions. Traditional prediction models, often limited by default parameters, have been superseded by more sophisticated approaches. Leveraging Bayesian optimization to fine-tune XGBoost, researchers can harness the power of complex data analysis to improve predictive accuracy. By identifying key factors influencing diabetes risk, personalized prevention strategies can be developed, ultimately enhancing patient outcomes. Successful implementation requires meticulous data management, stringent ethical considerations, and seamless integration into healthcare systems. This study focused on optimizing the hyperparameters of an XGBoost ensemble machine learning model using Bayesian optimization. Compared to grid search XGBoost (accuracy: 97.24%, F1-score: 95.72%, MCC: 81.02%), the XGBoost with Bayesian optimization achieved slightly improved performance (accuracy: 97.26%, F1-score: 95.72%, MCC:81.18%). Although the improvements observed in this study are modest, the optimized XGBoost model with Bayesian optimization represents a promising step towards revolutionizing diabetes prevention and treatment. This approach holds significant potential to improve outcomes for individuals at risk of developing diabetes.

## 1. Introduction

Diabetes is a chronic condition characterized by high blood sugar levels. It primarily manifests as Type 1 (immune system attacks insulin-producing cells) or Type 2 (insulin resistance or deficiency) [1–4]. Leveraging medical records, genetic data, and lifestyle information, machine learning can predict diabetes risk. By analyzing patterns within this data, machine learning algorithms, such as Neural Networks, Decision Trees (DT), and Logistic Regression (LR), can identify individuals likely to develop diabetes. Early detection empowers healthcare providers to implement lifestyle modifications or medical interventions, potentially delaying or mitigating the onset of the disease. While these technologies hold immense promise, ensuring responsible and sustainable use necessitates a focus on data quality, understanding predictive models, and adapting them to diverse healthcare settings.

Diabetes is a chronic condition characterized by elevated blood sugar levels, resulting from the body’s inability to produce or effectively utilize insulin. This metabolic disorder can damage vital organs, including the heart, blood vessels, and eyes [5]. Type 1 diabetes is an autoimmune disease where the body’s immune system attacks insulin-producing cells, while type 2 diabetes arises from insulin resistance or insufficient insulin production, often linked to lifestyle factors. Early detection and management are crucial to prevent severe complications [6]. Diabetes mellitus (DM) is a metabolic disorder characterized by persistently high blood sugar levels. These elevated glucose levels result from the body’s inability to produce or effectively use insulin, a hormone essential for regulating blood sugar. Chronic hyperglycemia can damage various organs, including the heart, blood vessels, eyes, kidneys, and nerves [7]. Diabetes mellitus, characterized by elevated blood sugar levels, has been recognized since ancient Egypt and India. The term "diabetes" originates from Greek, referring to excessive urination and the sweet taste of diabetic urine. In 1776,elevated blood sugar levels were first documented in Britain [8]. Early detection of diabetes is crucial for preventing complications. This study employs machine learning models to classify type 2 diabetes patients and identify the most effective model for predicting diabetes risk [9].

Elevated blood sugar, or hyperglycemia, is a metabolic disorder stemming from abnormalities in insulin production, insulin action, or both. Despite advancements in diabetes research, the definition of hyperglycemia remains unchanged. Chronic hyperglycemia disrupts carbohydrate, lipid, and protein metabolism, leading to damage and dysfunction in the cardiovascular, ocular, renal, arterial, and neural systems over time [10, 11] Diabetes is categorized by etiology and clinical presentation into Type 1, Type 2, and gestational diabetes. Type 1 diabetes results from an absolute insulin deficiency caused by the autoimmune destruction of pancreatic beta cells. Type 2 diabetes is characterized by insulin resistance and relative insulin insufficiency. Gestational diabetes, a glucose intolerance condition, develops during pregnancy. Less common forms of hyperglycemia can arise from medications, surgeries, genetic factors, inadequate nutrition, other disorders, and various circumstances [12, 13]. Type 2 diabetes is the most prevalent form, accounting for 90% of all diabetes diagnoses. While often diagnosed in individuals over 40 [14–16], Type 2 diabetes can affect younger people and children. Many cases are incidentally discovered during treatment for unrelated conditions, as symptoms may be absent for extended periods. Unlike Type 1 diabetes, individuals with Type 2 diabetes do not initially require insulin therapy. However, insulin may become necessary if blood sugar control cannot be achieved through diet or oral hypoglycemic medications alone.

The etiology of Type 2 diabetes is complex, involving multiple factors. While various risk factors influence disease occurrence, not all are direct causal agents [17–21]. These interconnected risks may be genetic, demographic (e.g., age), or behavioral. For instance, diet, smoking, obesity, and physical inactivity are notable behavioral risk factors, often termed "modifiable" due to their potential for change.

Type 2 diabetes is a rapidly increasing non-communicable disease with a global reach. The International Diabetes Federation reported over 460 million individuals with hyperglycemia in 2019, a figure projected to rise to 578 million by 2030 and exceed 700 million by 2045. The prevalence of approximately 4 million diabetics in Saudi Arabia [22] underscores the significance of this issue. Diabetes has severe health and economic consequences. Diabetics face a two to four times higher risk of heart disease and stroke. Type 2 diabetes frequently leads to chronic kidney damage, often necessitating dialysis or transplantation. The risk of lower limb amputation is increased 25-fold, and retinal degeneration can cause blindness. In 2019, diabetes and its complications claimed 4.2 million lives among individuals aged 20–79, resulting in minimum hospital costs of USD 760 billion. This figure is projected to increase to USD 825 billion in 2030 and USD 845 billion in 2045, representing 8.6% and 11.2% growth, respectively [23].

**Fig 1:**
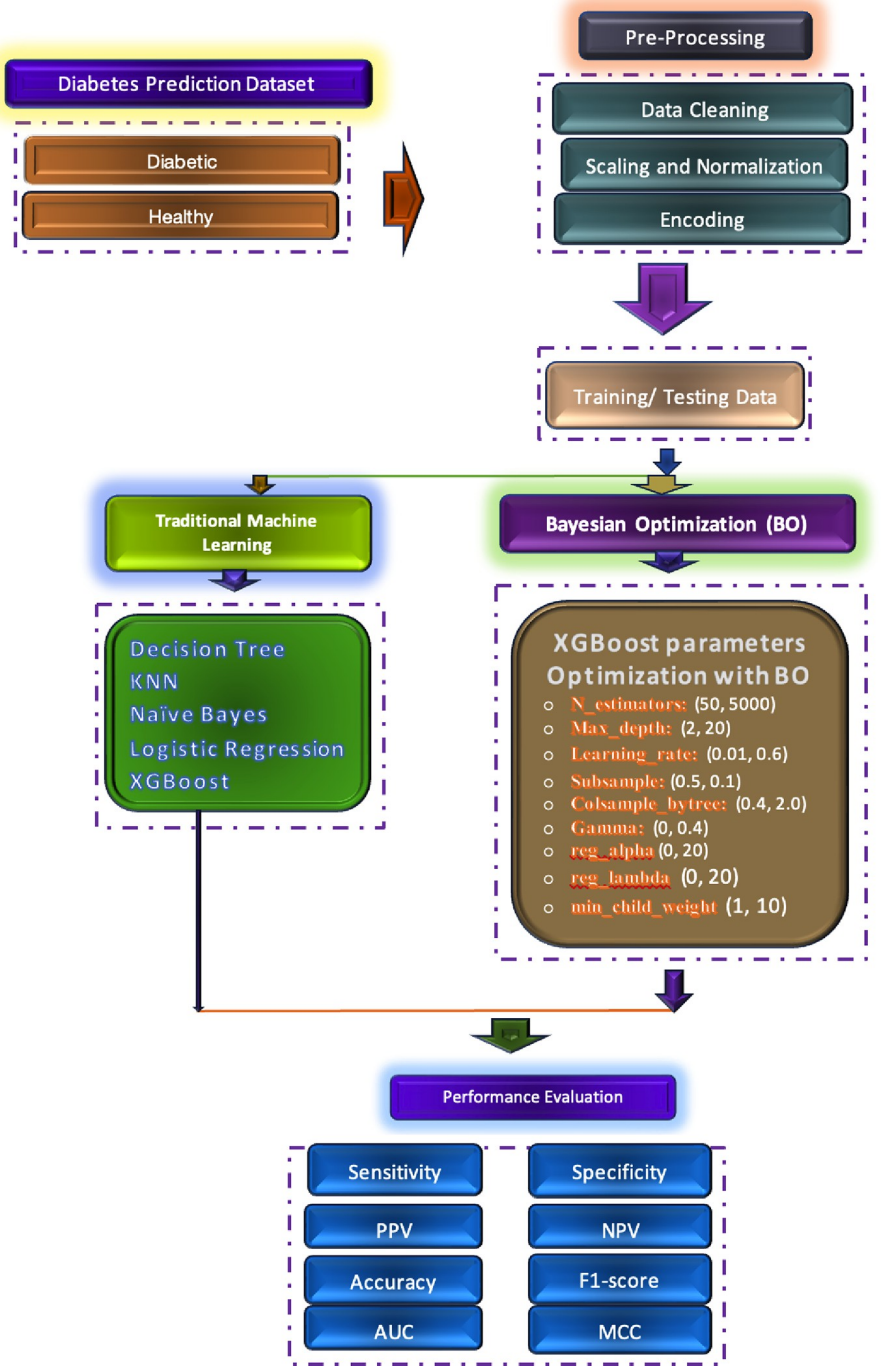
Schematic diagram to detect diabetes using optimized XGBoost with Bayesian optimization parameters selection.

Fig 1 outlines the methodological steps undertaken in this study to predict diabetes using a publicly accessible Kaggle dataset. The methodology includes three fundamental contributions:

1. To enhance data quality and model performance, a comprehensive preprocessing pipeline was implemented. This involved meticulous data cleaning, scaling, normalization, and encoding processes
2. To enhance XGBoost’s performance, we employed Bayesian optimization to meticulously tune its hyperparameters. This optimization process facilitated a comparative analysis against traditional machine learning algorithms, enabling us to assess the algorithm’s efficiency and effectiveness
3. A comprehensive model evaluation was conducted using a variety of metrics including AUC, PPV, NPV, accuracy, specificity, sensitivity, F1-score, and MCC. This multifaceted approach provides a more reliable assessment of model performance compared to solely relying on accuracy.

Further steps:

- **Data Splitting:** To rigorously evaluate model performance, the dataset was divided into training and testing subsets using five-fold cross-validation.
- **Model Comparison:** A comparative analysis was conducted between the optimized XGBoost model and established machine learning algorithms to assess its relative effectiveness and potential advantages

This study explores the efficacy of a meticulously preprocessed dataset in conjunction with an optimized XGBoost algorithm for enhancing diabetes prediction accuracy. Robust evaluation metrics will be employed to comprehensively assess the model’s performance.

The paper is structured as follows: Section one provides a foundational overview of diabetes, including its symptoms, causes, and associated challenges. The proposed model is also briefly introduced. Section two presents a comprehensive literature review, analyzing existing diabetes detection methods, their limitations, and the rationale for the proposed approach. Section three delves into the methodology, encompassing dataset description, traditional machine learning algorithms, and the novel XGBoost model, including its mathematical formulation. Section four presents the experimental results, visualized through bar graphs, scatter plots, and explainable AI techniques. Comparative analysis with traditional methods is conducted to highlight the proposed model’s strengths. The final section summarizes key findings, identifies study limitations, and outlines potential avenues for future research.

## 2. Related work

For many years, researchers have investigated diabetes prevalence and occurrence globally using diverse data and analytical methods [24–27]. Hyperglycemia, a metabolic disorder characterized by abnormal blood glucose levels, poses a significant challenge to public health in the 21st century [28–30]. In 2021, the global diabetes population reached 536.6 million, projected to increase to 783.2 million by 2045, imposing a substantial burden on healthcare systems [31]. Type 2 diabetes (T2DM) is the most prevalent form of the disease. Beyond predicting prediabetes, this study aids in identifying risk factors for diabetes development based on clinical data. Preventing diabetes involves comprehensive assessments of patient sociodemographic and health profiles, followed by tailored treatment plans addressing individual risk factors and comorbidities [32, 33].

The escalating prevalence of diabetes underscores the critical need for early diagnosis and effective prediction models. Given the disease’s severe consequences, research into diabetes prevention and prediction is imperative [34]. Numerous studies have explored diabetes etiology, identifying factors such as anthropometric characteristics (BMI), demographic variables (occupation), lifestyle factors (alcohol consumption), and genetics [35].

Machine learning algorithms have emerged as valuable tools for anticipating and diagnosing chronic diseases in public health. The global diabetes epidemic necessitates advanced methods for disease description, prediction, and evaluation [36, 37]. Supervised learning, encompassing Logistic Regression, K-Nearest Neighbors, Naive Bayes, Decision Trees, Artificial Neural Networks, and Support Vector Machines, is a widely employed machine learning technique [38–41].

Various neural network models, including Support Vector Machines, Back-Propagation Neural Networks, CART decision trees, and Deep Neural Networks, have been applied to type 2 diabetes risk prediction. However, comprehensive comparisons of these models’ predictive performance are lacking. A study utilizing Dongguan residents’ chronic disease risk factor data from 2016 to 2018 developed six diabetes risk prediction models: Logistic Regression, CART, BP Neural Network, SVM, and DNN. Comparative analysis of these models provides a methodological benchmark for type 2 diabetes prediction [42–44].

Artificial intelligence, particularly supervised machine learning, has proven invaluable in diabetes diagnosis and management. These models, built upon medical history, risk factors, and genetic data, demonstrate high accuracy in predicting diabetes development [45–47].

Machine learning (ML) research in diabetes prediction has explored numerous factors. As a subset of AI, ML enables software to predict events without explicit programming. Its ability to identify diabetes-related patterns within large datasets is crucial for model training. Unlike traditional statistical methods, ML effectively handles non-linear data. Medical studies have applied ML to predict hyperglycemia-related variables. By analyzing independent electronic health record variables, researchers have developed ML models for Type 2 diabetes prediction [48–50].

The American Heart Association (AHA) and the National Heart, Lung, and Blood Institute (NHLBI) have outlined clinical risk factors for ML-based diabetes prediction. Efforts to enhance diabetes prediction system performance using imbalanced data are ongoing. Comprehensive reviews indicate that while many studies have examined various factors for diabetes prediction, dietary factors are often overlooked [51–53]. ML models are also being employed to analyze medical images, such as CT and retinal scans, for early detection of diabetes and related conditions. As a relatively new field merging computer science and statistics, ML offers innovative solutions to complex problems. Researchers emphasize the need for developing tools to identify and address gaps in patient care [54–56]. These ML models aim to improve patient care quality while reducing healthcare costs. Accurately estimating the future economic impact of hyperglycemia is essential for healthcare policy development and cost management [57, 58]. Machine learning algorithms have become prevalent in public health for forecasting and detecting chronic diseases like diabetes. Numerous studies utilizing ML techniques, including SVM, ANNs, KNNs, and DT, have been conducted in diabetes modeling [59–61].

Beyond early diagnosis, machine learning holds the potential to revolutionize diabetes management. Envision personalized therapies based on risk profiles, insulin adjustments guided by predicted blood sugar fluctuations, and real-time portable devices supporting daily life [62–65]. Once considered futuristic, these scenarios are becoming increasingly feasible as researchers develop innovative approaches:

- Predictive Models: Utilizing data from continuous glucose monitors and other metrics, these models can forecast blood sugar spikes and drops, enabling timely interventions such as medication adjustments or dietary changes.
- Dynamic Implementation Methods: Adapting to an individual’s fluctuating blood sugar levels and overall health, these algorithms provide real-time medication dosage and lifestyle recommendations.
- Closed-Loop Insulin Delivery Systems: By predicting blood sugar trends and mimicking a healthy pancreas, these systems automatically adjust insulin delivery through integration with insulin pumps.

Ethical considerations remain paramount. Data biases can lead to discriminatory predictions, necessitating transparent algorithmic decision-making to foster trust. Preventing healthcare disparities and ensuring equitable access to these advanced tools are crucial.

The complex, nonlinear dynamics within diabetes patient datasets pose challenges for accurate detection and analysis. To address these complexities, this study employed Bayesian optimization to enhance the XGBoost algorithm by optimizing hyperparameters, surpassing the performance of conventional machine learning approaches.

XGBoost has emerged as a powerful tool for addressing complex medical challenges due to its predictive capabilities. Its applications extend to various medical domains, including the analysis of medical imaging data such as X-rays for diagnosis and prediction. The researcher [66] constructed a predictive model for metabolic syndrome by using Bayesian optimization and XGBoost, while integrating variables from traditional Chinese medicine (TCM). Prior research has examined many machine learning methods for forecasting metabolic syndrome, although only a limited number have included Traditional Chinese Medicine (TCM) characteristics. Indeed, [67] used logistic regression with decision trees, resulting in a modest level of accuracy. Support vector machines were used in a similar manner by [68], although their model did not include conventional diagnostic indications. The researcher [69] concentrated the prospect of integrating existing algorithms with conventional medical knowledge; however, their methodology excluded Bayesian optimized performance. The authors [70] exhibited the efficacy of XGBoost in medical prognostications, but without concentrating on metabolic syndrome. The researchers [71] showed the significance of feature selection in enhancing the effectiveness of models, which is compatible with the present study’s use of Bayesian optimization. The authors [72] also studied the inclusion of Traditional Chinese Medicine (TCM) into mathematical models for prediction, however their research could not achieve the same degree of precision as that of [73]. The simulation results indicated that the model provided in this study attained values of 93.35%, 90.67%, 80.40%, and 0.920 for the F1, sensitivity, FRS, and AUC measures, accordingly. The findings rivalled the efficiency of the seven other machine learning models that had been examined. In conclusion, this research has created a smart forecasting application for MetS using the suggested model. This application can be accessed by regular users who can then conduct self-diagnosis by completing a web-based questionnaire. The main goal of this application is to identify and intervene in MetS at an early stage.

The researchers [74] established a hybrid model to enhance the safety of IOT networks. They were accomplished this through integrating XGBoost with Convolution Neural Networks (CNNs) and improving the model employing an enhanced reptilian searching methods. The past study examined various approaches to ensure privacy in the Internet of Things (IoT). The authors [75] employed modern deep learning algorithms for identifying anomalies, contributing to substantial enhancements in reliability. The main goal of [76] research was to improve machine learning models’ efficiency in the context of Internet of Things cybersecurity. Even so, the strategies they employed were different from the hybrid methodology observed in the study carried out by [74]. The researchers [77] examined the use of algorithmic development to enhance hyper parameters. (Zivkovic et al., 2022) pointed out the crucial role of feature extraction in improving the model performance, harmonizing with the convolutional neural networks (CNN) component addressed by [74]. However, their research did not attain the same degree of optimum. The authors [78] illustrated the need of adaptable and flexible security solutions in Internet of Things networks, a requirement that is met by [74] hybrid architecture (2023). The authors [79] examine the forecasting of stress levels among healthcare employed within the COVID-19 pandemic using sophisticated machine learning algorithms. The analysis implemented XGBoost, SHAP values, and a tree model to examine data from 436 healthcare practitioners in North India. The findings indicate that 52.6% of the participants encountered significant mental illness. Notable emphasized that have been identified include shortages of medication and difficulties in maintaining focus. The XGBoost algorithm exhibited an accuracy of 88% in predicting stress levels, demonstrating the significant influence of the global epidemic on the psychological health of healthcare professionals and emphasizing the need for specific treatments to address these stress factors.

The researchers [80] conducted a study to proposed an innovative technique utilizing chest X-ray pictures that allowed quickly detecting COVID-19. The integration of Convolution Neural Networks (CNN) with Extreme Gradient Boosting (XGBoost) approaches was included in the suggested method. For feature extraction a Convolution Neural Network (CNN) was used with the XGBoost algorithm to do the classification. By implementing an enhanced Arithmetic Optimizations Algorithm (A.O.A) the hyper parameters of XGBoost had been optimized. The hybrid technique was attained a classification accuracy of 99.39%, a weighted average precision of 0.993889, an F1-score of 0.993887, and a recall of 0.993887. Using a large dataset of 12,000 X-ray pictures the accuracy of the model was evaluated. The viral pneumonia, normal and COVID-19 images were classified into three distinct categories. The techniques were demonstrated higher accuracy in comparison to existing cutting-edge techniques, making it a compelling tool for rapid identification of COVID-19.

Traditional diabetes detection methods often rely on machine learning algorithms with default settings and limited data preprocessing. This study introduces a refined approach that incorporates robust data preprocessing techniques to enhance data quality. Subsequently, an XGBoost ensemble model is employed and optimized using Bayesian hyperparameter tuning to effectively address the complexities of diabetes prediction. This novel methodology surpasses traditional grid search and random search techniques, leading to improved diabetes detection performance. The proposed optimized XGBoost model significantly outperformed other methods in predicting diabetes, demonstrating its potential for early disease detection. This superior performance holds promise for improving diagnostic accuracy and treatment planning, ultimately leading to better outcomes for individuals at risk of diabetes.

## 3. Materials and methods

### 3.1. Dataset

The publicly available Kaggle dataset on diabetes prediction offers a valuable resource for both healthcare professionals and researchers. Comprising medical and demographic data from patients, including diabetes status, age, gender, BMI, and various health conditions, the dataset facilitates the development of machine learning models capable of predicting diabetes risk. By identifying at-risk individuals proactively, healthcare providers can implement tailored prevention and treatment strategies. Researchers can leverage this data to uncover underlying patterns and relationships between medical factors and diabetes, contributing to the advancement of diabetes prevention and management.

### 3.2. Machine learning algorithms

To optimize diabetes prediction, we explored a variety of supervised machine learning classification algorithms, with a particular emphasis on the optimized XGBoost model. Ensemble methods, which combine multiple models to outperform individual ones, are a powerful tool in machine learning [81–84]. By strategically combining diverse models, ensembles create a single, robust predictor. Extensive research confirms the superior accuracy of ensemble methods, even with varying model complexities. Boosting, a prominent ensemble technique, iteratively constructs strong classifiers from weak ones [85–87]. Through repeated sampling of training data, boosting converges on a robust final model. To conduct a thorough evaluation, we employed a range of carefully optimized machine learning algorithms. The specific methodologies used are detailed in the following sections.

#### 3.2.1. Decision Tree (DT)

In the discipline of AI, DTs are sophisticated computational models which belong to the CARTs class [88–90]. Breiman developed these types of algorithms in 1984, which are extremely efficient in comprehending the complicated associations between input factors and a target variable, producing robust forecasting perspectives. DTs proceed through an ordered hierarchy of decision phases, everyone carefully evaluating an individual predictive variable, analogous to a tree splitting into multiple routes. For the objective variable at each final endpoint, they are capable to develop the prediction following a systematic evaluation of these characteristics. This type of approach is extremely helpful when coping with enormous and complicated datasets, performing as an effective decision supporting instrument throughout the fields of machine learning and data mining. To evaluate and analyzing the DT’s primary component procedures which is a comprehensive method. This research study data points categories and precisely evaluates the dataset by Finding patterns or regularities. As the decision tree models DT’s employed efficiently and by identifying specific features to classify data that optimize data partition [91]. When their certain threshold requirement is achieved through the method described here organizes attributes into specific categories. By revealing data patterns and relationships the method Decision Trees (DTs) enhance predictive modeling and decision-making then the method is easy to comprehend.

DT algorithm can be written algebraically as expressed in Eqs 1–3::

$$\bar{X}=\{X_{1},X_{2},X_{3},,\ldots \ldots ..,X_{m}{\}}^{T}$$ (1)

$$X_{i}=\{x_{1i},x_{2i},x_{3i},,\ldots ,,\ldots ..,x_{ini}\}$$ (2)

$$S=\{S_{1i},S_{2i},,\ldots S_{ii}\ldots \ldots \ldots .S_{mi}\}$$ (3)

In the following mathematical algebraic expressions, the mathematical notations are used as follows:

- m: Indicates all numbers of data points within the entire dataset.
- n: Indicates the number of independent variables being considered.
- S: This denotes an m-dimensional vector, encompassing the values of the variable you intend to forecast.
- $X_{i}$: Symbolizes the ith module within an n-dimensional vector of autonomous variables.
- $\bar{X}$: Represents the comprehensive pattern vector comprised of all autonomous variables.
- T: The symbol T denotes the transposition operation, commonly used to transform a row vector into a column vector or vice versa.

To elucidate further, the equations depict a relationship between the variable you aim to forecast (represented by S) and a collection of independent variables (encapsulated within X). The number of observations within your dataset dictates the dimensionality of S, while the quantity of independent variables shapes the dimensionality of X.

#### 3.2.2. k- Nearest-Neighbors (KNN)

Additionally discipline of detection of patterns the technique which is the KNN is an extremely versatile tool. It can be utilized to solve regression complications as well as classification, while assuming certain restricted predictions about the fundamental data distribution [92–95]. The basic concept of this method is to identify the k most identical the instances in the dataset used for training by calculating actual Euclidean distances. The additional information can be identified and categorized through utilizing the combined features of these examples.

Throughout an assortment of consecutive steps, the method efficiently proceeds as:

1. Exploration of feature space: Systematically maintaining the relationships between data values the tool consistently meticulously draws the characteristic space.
2. Calculation of The distance: It estimates its prospective neighbors in the training dataset and Euclidean distance between each new data point precisely.
3. Arrangement of the Neighborhood: The technique which rigorously classifies the respective distances in the ascendant order therefore revealing the nearest neighbors.
4. Making the Decision: KNN utilizes the weighted average or a traditional voting system in order to make predictions depending upon whether the method is applied to regression or classification tasks respectively.
5. The modification is meticulously generated according to the characteristics and the amount of data and the effectiveness of method is impacted by the numbers of neighbors (k). The smoother decision boundaries that are produced with higher values for larger datasets.

In the area of study, the most appropriate value of k identifying is a particularly captivating issue requires usually extensive testing along with expertise. As for the particular choice there are no developed statistical techniques currently but by implementing the random value and then gradually enhancing it through the investigation and evaluation that mostly produces desirable outcomes. As for the classification and regression problems the KNN method provides a very versatile and adaptive method and without imposing strict assumptions it is very adapting to various data domains. Its ability to learn directly from the training data, without the need for complex model building, further enhances its appeal and practicality in various real-world applications.

#### 3.2.3. Naïve Bayes (NB)

In Bayesian classification the NB algorithm is an essential technique within the discipline of machine learning. It has its foundation on Bayes’ theorem and is frequently seen as an essential component of this discipline [96, 97]. In 1963 Masteller and Wallace revealed in the beginning among all practitioners it has gracefully retained its position as an ideal mechanism due to its remarkable versatility and computational efficiency.

The basic foundation focuses on an essential assumption: The feature’s independent condition. It frequently produces extremely powerful results in an extensive variety of everyday scenarios whereas the premises can seem implausible. Fundamental attributes of NB are as follows:

- Effortless handling of high-dimensional data: NB gracefully navigates datasets with numerous features, making it a valuable ally in domains like text classification and spam detection.
- Accommodation of diverse feature types: It effortlessly embraces both categorical and continuous variables, enhancing its adaptability to various problem settings.
- Superior computational efficiency: NB constructs its model with remarkable swiftness, making it a practical choice for time-sensitive applications.
- Demonstrated effectiveness in numerous domains: Its proven track record across various fields, often outperforming algorithms like decision trees, C-means, and SVMs, solidifies its reputation as a reliable classification workhorse.

Naïve Bayes (NB) depends upon the features of independence that can offer increase to errors. On the other hand, several solutions exist which can minimize these biases and maintain its effectiveness. The complicated relation that exists between variance and bias in contrast attempts to directly eliminate probabilistic errors in calculation frequently offer contrary to the results.

#### 3.2.4. Optimized XGBoost

With the utilization of proficient methodologies achieves optimum performance XGBoost, a born champion [98, 99]. To enhance the efficiency XGBoost employs several approaches, such as fine-tuning its underlying parameters and individually optimizing its decision trees. Without afflicted by excessive fitting the regularization process promises that the algorithm develops whereas attentive trimming maintains its effectiveness [100–103]. Utilizing multiple priorities to accomplish efficient development it succeeds in interaction. But optimizations extend beyond its internals. Feature engineering polishes the data it feeds on, while advanced boosting techniques enhance its learning power. And if that’s not enough, it can even adjust its approach to exploit specific hardware strengths. Constant development and a focus on both elegance and efficiency keep XGBoost at the forefront of machine learning.

Emerging in 2016, the XGBoost system, proposed by Chen and Guestrin, quickly rose to prominence within the machine learning landscape [104–106]. By leveraging the power of gradient boosting, it established itself as a leading tool for tackling supervised learning challenges, exceeding the performance of many established methodologies. At its core, XGBoost builds upon the concept of ensemble learning, seamlessly combining weaker base models into a progressively stronger learner through an iterative approach.

In this study, we utilized XGBoost’s capabilities by employing a combination of linear and tree-based models, further enhanced through strategic optimization parameters. These parameters were carefully chosen to address the intricacies of the optimization problem within the context of gradient boosting, effectively tailoring the step direction and step size for optimal model performance as Eq (4):

$$\frac{\partial Z_{l}(x,f^{l-1}(y)+f_{l}(y)}{\partial f_{l}(y)}=0$$ (4)

For each ‘y’ in data to directly fix the step we have Eqs 5–7:

$$Z_{l}(x,f^{l-1}(y)+f_{l}(\left(y\right)),$$ (5)

$$\approx Z_{l}(x,f^{l-1}(y))+g_{l}(y)f_{l}(y)+\frac{1}{2}h_{l}(y)f_{l}{\left(y\right)}^{2},$$ (6)

$$\approx Z_{l}(x,f^{l-1}(y))+g_{l}(y)f_{l}(y)+\frac{1}{2}h_{l}(y)f_{l}{\left(y\right)}^{2}.$$ (7)

Utilizing the 2nd order Taylor series expansion by expending loss function, where *g*_ℓ_(*y*) is gradient and *h*_ℓ_(*y*) is Hessian as reflected in Eq 8.


$$h_{l}(y)=\frac{\partial^{2}Z_{l}(x,f(y))}{\partial f{\left(y\right)}^{2}},heref(y)=f^{l-1}(y).$$ (8)

Then, loss function can be rewritten as Eqs 9 & 10:

$$Z_{l}(f_{l})\approx \sum_{i=1}^{m}[g_{l}(y_{i})f_{l}(y_{i})+\frac{1}{2}h_{l}(y_{i})f_{l}y^{2}]+Constant,$$ (9)

$$\propto \sum_{t=i}^{p_{l}}\sum_{t\in R_{tl}}[g_{l}(y_{i})L_{tl}+\frac{1}{2}h_{l}(y_{i})L_{tl}^{2}].$$ (10)

In region t, let’s G_tn_ denotes sum of gradient and the sum of Hessian is represented by H_tn_ then equation will be as indicated in Eq 11,

$$Z_{l}(f_{l})\propto \sum_{t=i}^{p_{l}}[G_{tl}L_{tl}+\frac{1}{2}H_{tl}L_{tl}^{2}].$$ (11)

The maximum value can be obtained by utilizing the following below function in Eq 12:

$$L_{tl}=-\frac{G_{tl}}{H_{tl}},Wheret=1,2,\ldots \ldots ..p_{n}.$$ (12)

We get loss function when we plug it back in Eq 13:

$$Z_{l}(f_{l})\propto -\frac{1}{2}\sum_{t=1}^{p_{l}}\frac{{G^{2}}_{tl}}{H_{tl}}.$$ (13)

This function is used to indicate the tree structure. A lower score suggests a more optimal structure (Chen and Guestrin 2016). The maximum advantage for each division is refleted in Eq 14:

$$Gain=\frac{1}{2}\left[\frac{G_{tlLeft}^{2}}{H_{tlLeft}}+\frac{G_{tlRight}^{2}}{H_{tlRight}}-\frac{G_{tl}^{2}}{H_{tl}}\right],$$ (14)

which is indicated in Eq 15,

$$Gain=\frac{1}{2}\left[\frac{G_{tlLeft}^{2}}{H_{tlLeft}}+\frac{G_{tlRight}^{2}}{H_{tlRight}}-\frac{{\left(G_{tlLeft}+G_{tlRight}\right)}^{2}}{H_{tlLeft}+H_{tlRight}}\right],$$ (15)

In order to enhance the performance, the loss function might be reformulated while considering the periodicity parameters refected in Eqs 16 and 17:

$$Z_{l}(f_{l})\propto \sum_{t=1}^{p_{l}}[G_{tl}L_{tl}+\frac{1}{2}H_{tl}L_{tl}^{2}]+\alpha P_{l}+\frac{1}{2}\mu \sum_{j=1}^{p_{l}}L_{tl}^{2}+\beta \sum_{j=1}^{p_{l}}\left|L_{tl}\right|$$ (16)

$$=\sum_{t=1}^{p_{l}}\left[G_{tl}L_{tl}+\frac{1}{2}(H_{tl}+\mu )L_{tl}^{2}+\beta |L_{tl}|\right]+\alpha P_{l},$$ (17)

Where "α" penalizes the number of leave, "β" represents L_1_ regularization, and "μ" represents L_2_ regularization. The optimum weight for each area j can be calculated as computed in Eqs 18 and 19:

$$L_{tl}=\left\{\begin{array}{ll} -\frac{G_{tl}+\beta }{H_{tl}+\mu } & G_{tl}<-\beta \\ -\frac{G_{jn}-\beta }{H_{tl}+\mu } & G_{tl}>\beta \\ \;0 & \mathrm{else} \end{array}\right\}$$ (18)

And the Gain is,

$$Gain=\frac{1}{2}\left[\frac{P_{\beta }(G_{tlLeft}^{2})}{H_{tlLeft}+\mu }+\frac{P_{\beta }(G_{tlRight}^{2})}{H_{tlRight}+\mu }-\frac{P_{\beta }{\left(G_{tl}\right)}^{2}}{H_{tl}+\mu }\right]-\alpha ,$$ (19)

Where,

$$P_{\beta }(G_{g})=\left\{\begin{array}{c} {G_{g}+\beta \;G}_{g}-\beta \\ G_{g}-\beta \;G_{g}\beta \\ 0\;\mathrm{else} \end{array}\right\}$$

#### 3.2.5. XGBoost algorithm

**Step 1.** Preparation:

- Gather and preprocess the data to ensure it’s suitable for modeling.
- Classify the data set in the form of testing sets and training sets to predict the model efficiency.

**Step 2**. Initialization:

- Start with a simple initial model, often a single decision tree with a constant prediction.

**Step 3**. Iterative Improvement Loop:

- Repeat the following steps until a stopping criterion is met:
  - Calculate Gradients:—Evaluate how far off the current model’s predictions are from the true labels in the training data.—Calculate a "gradient" for each data point, indicating the direction and magnitude of necessary correction.
  - Train a New Tree:—Build a new decision tree, focusing on areas where the model is making the largest errors (based on the gradients).—The tree learns to "fit" these gradients, aiming to correct the model’s mistakes.
  - Add to Ensemble:—Incorporate the new tree into the existing ensemble model, assigning it a weight based on its performance.—The model’s prediction now becomes a weighted combination of the predictions from all trees.
  - Regularize:—Employ regularization techniques to prevent overfitting:—L1/L2 regularization: Penalize model complexity to encourage simpler trees.—Shrinkage: Scale down the contribution of each new tree to promote cooperation and avoid over-reliance on individual trees.—Column subsampling: Randomly choose the subdivision of characteristics for every tree to increase diversity and reduce overfitting.

**Step 4**. Tree Pruning

- Simplify trees by removing non-essential branches or nodes, potentially improving efficiency and reducing overfitting.

**Step 5**. Final Prediction:

- Once the stopping criterion is met (e.g., desired accuracy attained), use the trained ensemble model to make predictions on new data:
- Each tree in the ensemble provides its prediction for the new data point.
- The final prediction is calculated as a weighted average of these individual tree predictions.

#### 3.2.6. Parameters optimization with Bayesian optimization

Bayesian optimization excels over traditional methods like grid and random search in optimizing XGBoost hyperparameters. It constructs a probabilistic model to efficiently explore the hyperparameter space, focusing on promising regions. This approach accelerates convergence to optimal solutions while reducing computational costs. By effectively balancing exploration and exploitation, Bayesian optimization adapts its search based on previous results, increasing the likelihood of finding optimal hyperparameters. Moreover, its ability to handle complex objective functions, incorporate prior knowledge, and be less sensitive to initial conditions solidifies its advantage. Consequently, Bayesian optimization emerges as a preferred choice for hyperparameter tuning in machine learning due to its efficiency, intelligence, and adaptability.

Under the hood of Bayesian optimization (BO) lies a treasure trove of technical techniques that propel its efficiency and power [100, 101, 107, 108]. Acquisition functions like Expected Improvement (EI) and Upper Confidence Bound (U.C.B) guide exploration & exploitation, while Gaussian Process Regression (GPR) and Kriging models act as probabilistic maps of the optimization landscape [109, 110]. These models are continuously updated through Bayesian update methods like MCMC and variational inference, reflecting new data and leading us closer to the peak. Strategies like batch BO and multi-armed bandit setups further enhance performance. Compared to brute force or traditional methods, BO is like a seasoned cartographer with a dynamic map, navigating the uncertain terrain of complex functions with finesse and efficiency. Its technical arsenal gives it the edge, pushing the boundaries of optimization and reaching the summit of your goals faster and more reliably.

XGBoost’s performance is significantly influenced by a subset of its hyperparameters. Key factors include tree complexity (max_depth, min_child_weight), learning rate (eta), and the number of trees (n_estimators). Regularization parameters (gamma, reg_alpha, reg_lambda) help prevent overfitting. Additionally, controlling the proportion of data used for training each tree (subsample, colsample_bytree) is crucial for model generalization.

**Step 1.** Import necessary libraries:

- Bring in the XGBoost library for building the model.
- Import the Bayesian optimization functionality for hyperparameter tuning.
- Import the procedure to split the dataset into training and testing sets.

**Step 2**. Define the hyper-parameter grid:

param_grid = {

’n_estimators’: (50, 5000),

’max_depth’: (2, 20),

’learning_rate’: (0.01, 0.6),

’subsample’: (0.5, 1.0),

’colsample_bytree’: (0.4, 2.0),

’gamma’: (0, 0.4),

’reg_alpha’: (0, 20),

’reg_lambda’: (0, 20),

’min_child_weight’: (1, 10),

}

**Step 3.** Create an XGBoost classifier:

- Instantiate an XGBoost classifier with a specified objective function and random state.

**Step 4.** Set up Bayesian optimization:

- Create a Bayesian optimization object, providing:
  ○ The XGBoost classifier to tune.
  ○ The hyperparameter grid.
  ○ The number of iterations (100).
  ○ The number of cross-validation folds (5).
  ○ A random state for reproducibility.

**Step 5.** Fit the model (with correction):

- Train the model using the training data (corrected from using testing data).

### 3.3. Performance evaluation measures

The performance of the proposed system for diabetes detection is evaluated using PPV, NPV, specificity, sensitivity, and overall accuracy [111].

TP: Accurate identification of abnormalities

FP: Incorrect identification of abnormalities.

TN: Accurate identification of normal cases.

FN: Incorrect identification of normal cases

Confusion Matrix

A confusion matrix, a commonly used tabular representation, is employed in this research to evaluate the performance of our classification model on the test dataset. While this method is straightforward to comprehend, the associated terminology can be perplexing. Its effectiveness is determined by comparing predicted outcomes to known true positive and true negative values.

#### 3.3.1. Sensitivity

Sensitivity is a metric that assesses a classifier’s ability to correctly identify positive cases. It represents the probability of a positive test result for a patient with the disease and is also known as the True Positive Rate (TPR). The Eq 20 express mathematically:

$$Sensitivity=\frac{TP}{TP+FN}$$ (20)

#### 3.3.2. Specificity

Specificity measures a classifier’s ability to correctly identify negative cases. It calculates the proportion of true negative instances out of all actual negative cases. Also known as the True Negative Rate (TNR), specificity is defiend in Eq 21

$$Specificity=\frac{TN}{TN+FP}$$ (21)

#### 3.3.3. Positive Predictive Value (PPV)

The PPV is a measure of how likely it is that a person who tests positive for a disease actually has the disease. In other words, it represents the proportion of positive test results that are true positives. Mathematically, in Eq 22.


$$PPV=\frac{TP}{TP+FP}$$ (22)

#### 3.3.4. Negative Predictive Value (NPV)

The NPV indicates the probability that a person who tests negative for a disease truly does not have the disease. It essentially measures the accuracy of negative test results, Mathematically, in Eq 23.


$$NPV=\frac{TN}{TN+FN}$$ (23)

#### 3.3.5. Accuracy

The Accuracy is a metric used to evaluate the overall performance of a classification model. It represents the proportion of correct predictions made by the model out of the total number of predictions. The Eq 24 defined it mathematically.


$$Accuracy=\frac{TP+TN}{TP+FP+FN+TN}$$ (24)

#### 3.3.6. F1 score

F1 scores provide a balanced assessment of a classifier’s performance by combining precision and recall into a single metric. They are calculated as the harmonic mean of these two values, mathematically, Eq 25 express it

$$F1-score=\frac{2*(Accuracy*Recall)}{Accuracy+Recal}$$ (25)

#### 3.3.7. Matthews Correlation Coefficient (MCC)

MCC comprehensively evaluates classifier performance by considering all elements of the confusion matrix. This makes it particularly robust for datasets with imbalanced class distributions. Mathematically, Eq 26 define it:

$$MCC=\frac{\left(TP*TN-FP*FN\right)}{\sqrt{\left(TP+FP)*(TP+FN)*TN+FP)*TN+FN\right)}}$$ (26)

### 3.4. Area under the ROC Curves (AUC)

The AUC quantifies a classifier’s ability to distinguish between positive and negative classes across various classification thresholds. It is calculated by plotting True Positive Rates (TPR) against False Positive Rates (FPR) at different threshold settings.

## 4. Results and discussions

This study initially employed traditional machine learning algorithms with default parameters for diabetes prediction. Subsequently, the performance was enhanced by optimizing the XGBoost ensemble model using grid search and Bayesian optimization techniques.

**Fig 2:**
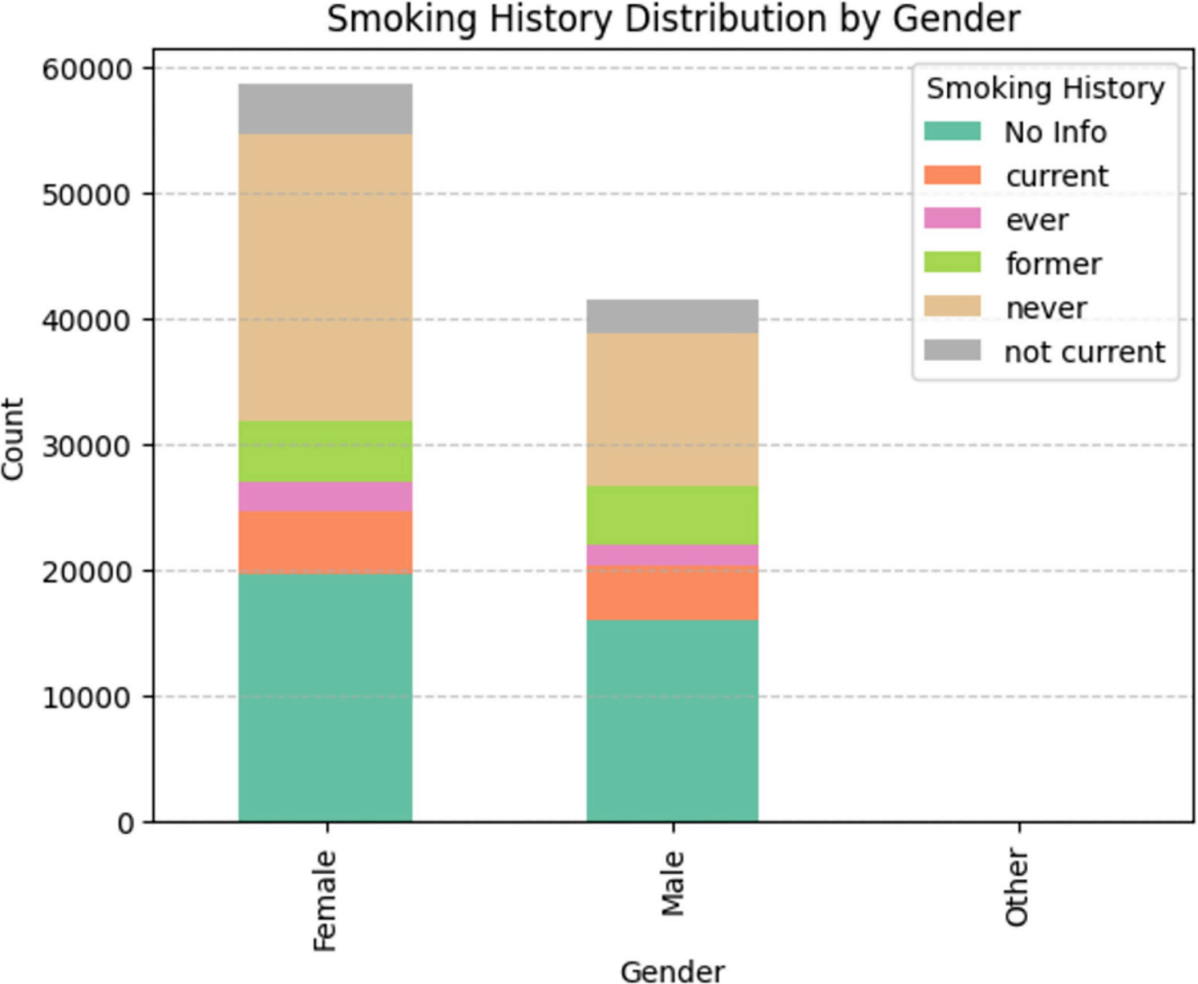
Smoking history distribution.

**Fig 2** illustrates the distribution of smoking history among participants. A total of 35,095 individuals never smoked,9,352 were former smokers, 9,286 were current smokers, and 4,004 had unknown smoking status. The figure further breaks down smoking history by gender.

Among females, 19,700 had no smoking information, 5,058 were current smokers, and 7,296 had ever smoked (including 5,058 current smokers and an unspecified number of former smokers). For males, corresponding figures were 16,110 with no information, 4,228 current smokers, and 5,993 ever smokers (including 4,228 current smokers and an unspecified number of former smokers). Additionally, 22,869 females and 12,223 males never smoked.

**Fig 3:**
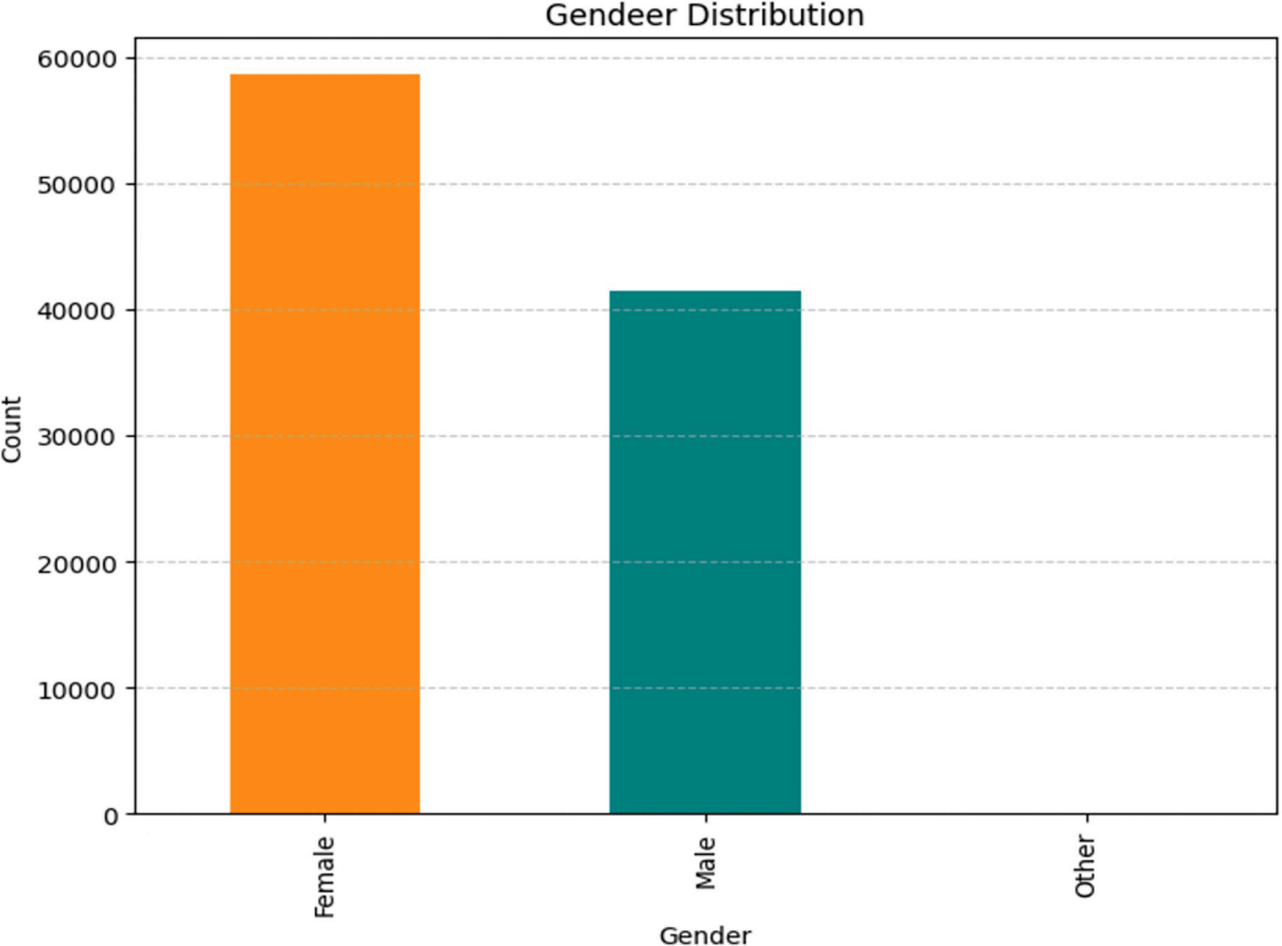
Gender based distribution.

Fig 3 depicts the gender distribution of the sample population, consisting of 5852 females, 41,430 males, and 18 individuals identifying as other genders, for a total of 100,000 participants.

### 4.1. Statical analysis

To differentiate diabetic and non-diabetic patients, a Chi-square test was conducted. The resulting test statistic of 141.60 and p-value of 1.188e-32 indicate a highly significant difference between the two groups. A similar chi-square test was performed to assess the relationship between the outcome variable and categorical predictors, revealing a significant association. Subsequent analysis will focus on numerical variables.

**Fig 4:**
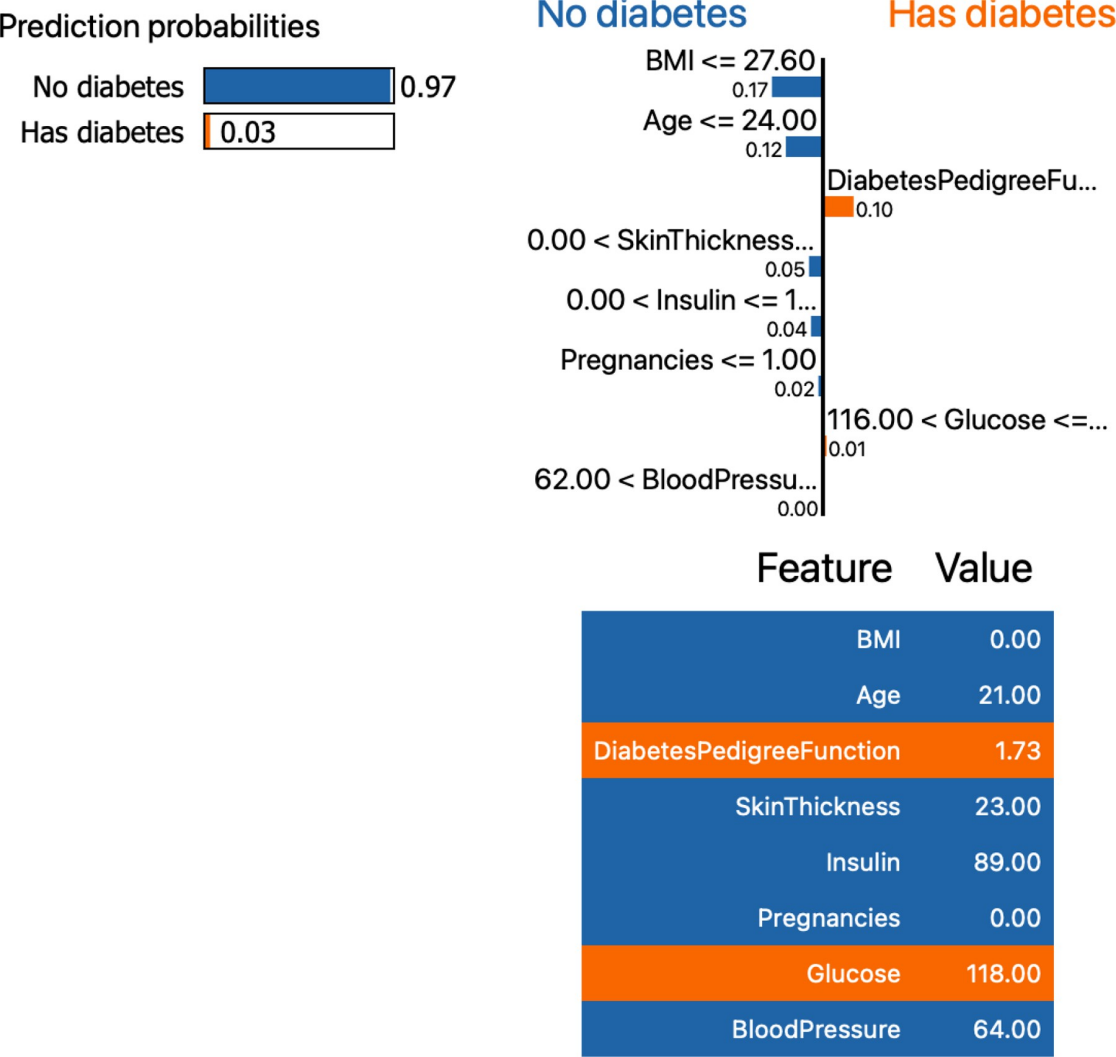
Prediction probabilities.

The Fig 4 reflects the prediction probabilities of features for no diabetes and with diabeties.

**Fig 5:**
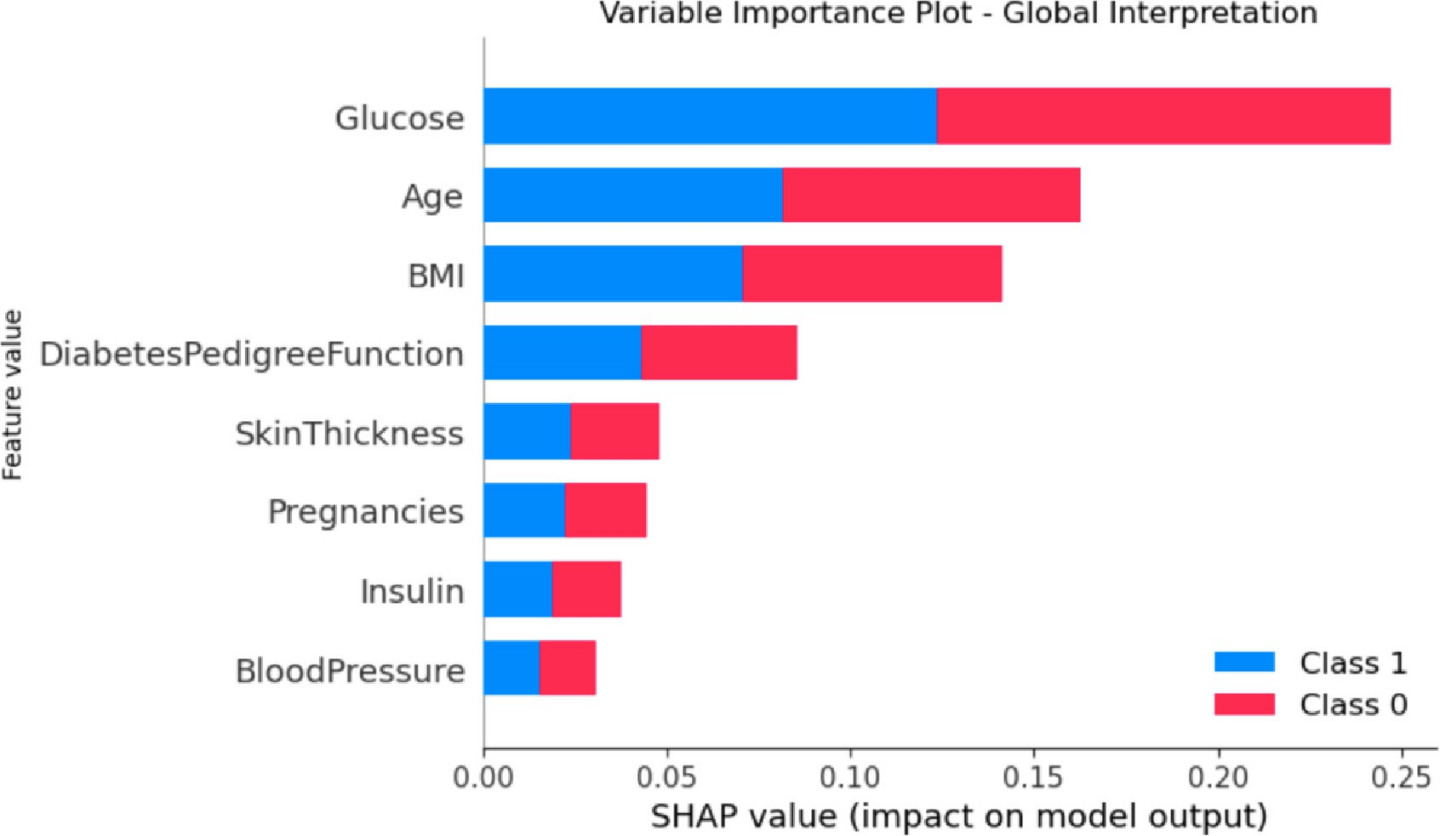
Feature importance.

The Fig 5 reflect the feature importance of diabetes prediction with class 0 and 1 of selected features. The Glucose feature has higher feature importance value followed by Age and so on.

**Fig 6:**
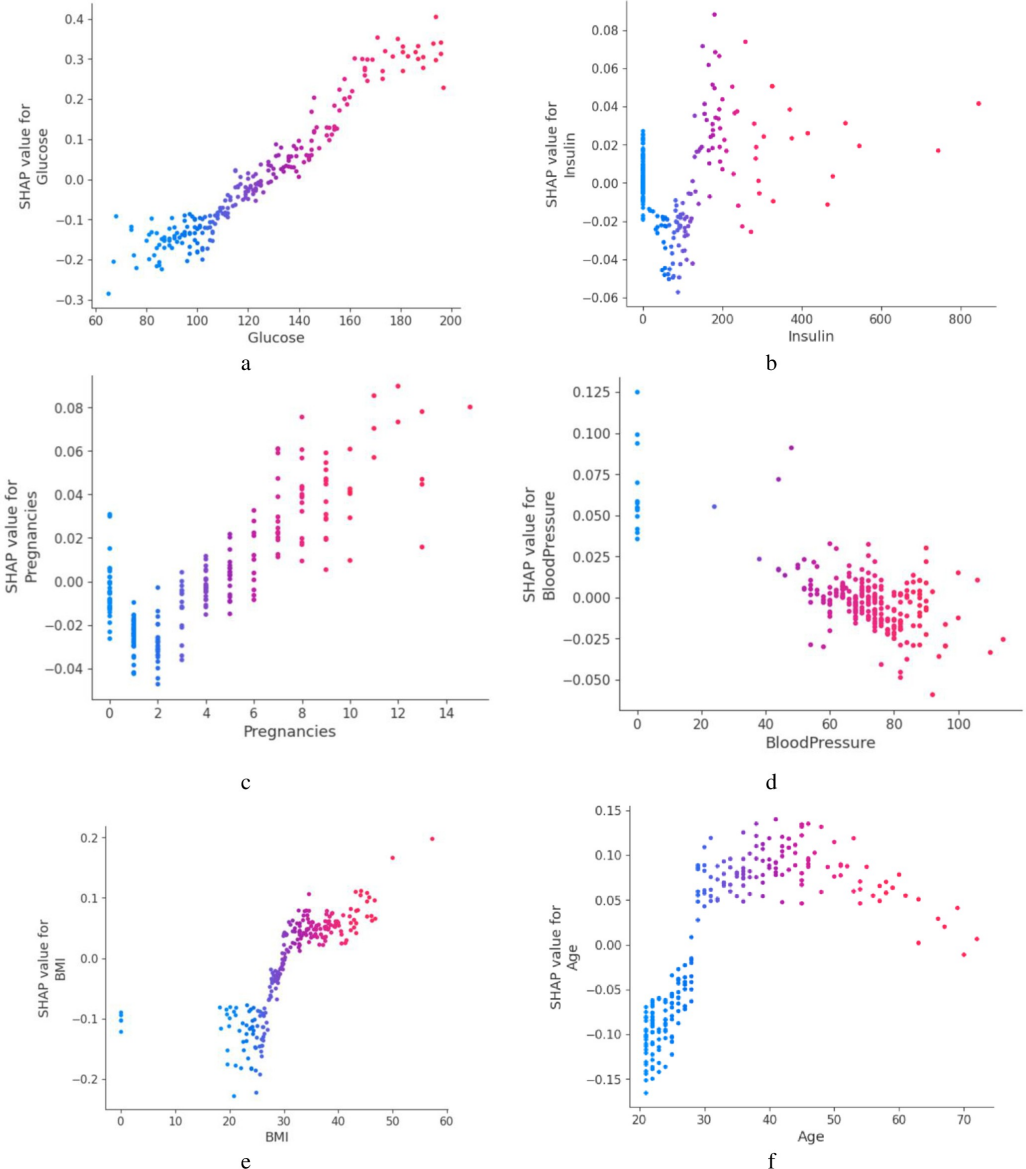
Scatter Plot of selected Variables a) Glucose, b) Insulin, c)Pregnancies, d) Blood Pressure, e) BMI, f) Age.

The Fig 6 reflects the sampels of scatter graph of both classes of diabetes and no-diabetes of selected features incuding Glucose, Insulin, Pregnanceis, BloodPressure, BMI, and age.

**Fig 7:**
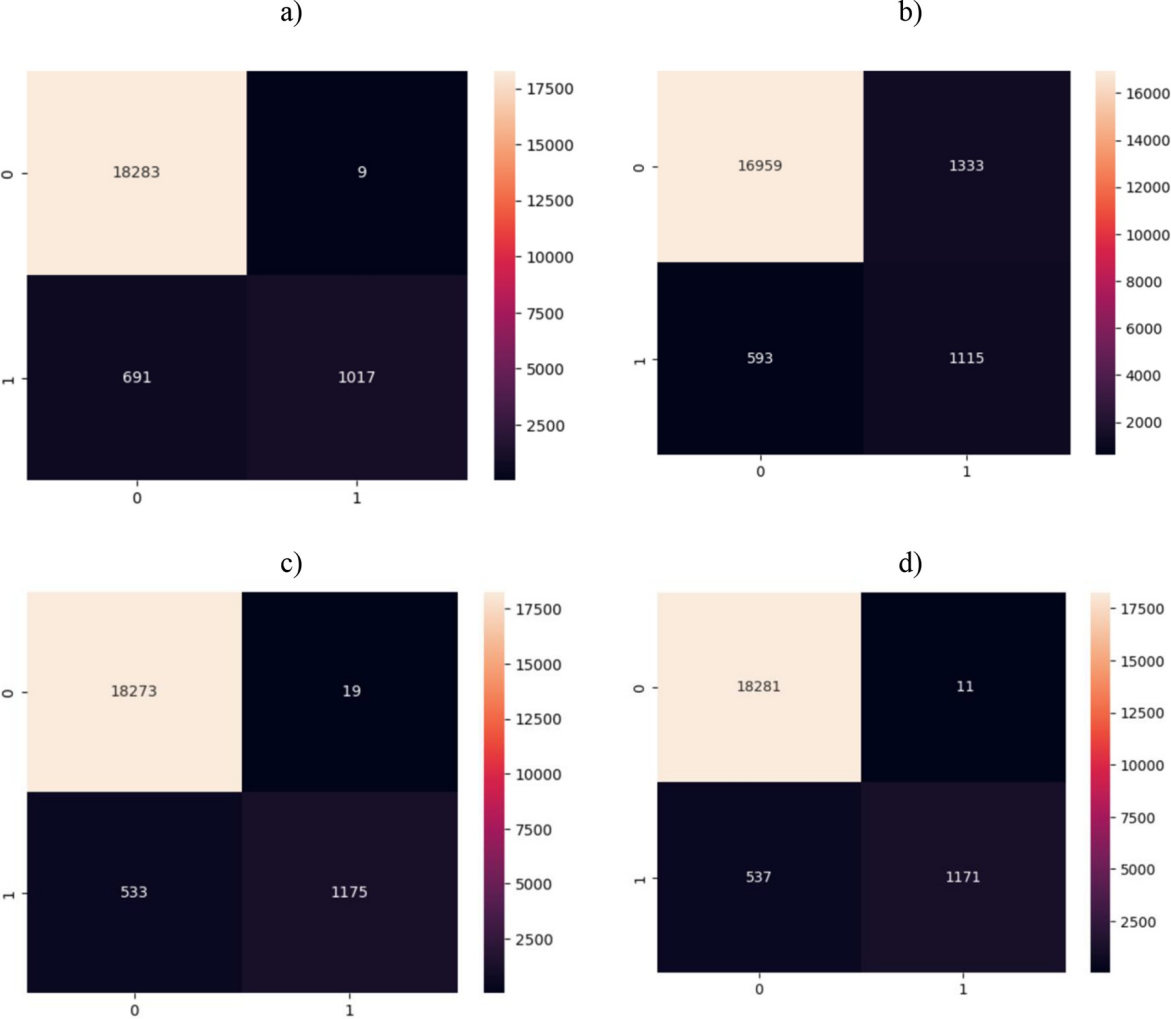
Confusion matrix a) SVM, b) XGB Fraud detection with optimized parameters, c) XGB parameters optimization with grid search, d) XGB parameters optimization with Bayesian optimization.

The Fig 7 presents the distribution of diabeters vs no diabetes of actual and predicted classes using confusion matrix by employing different machine learning algorithms. Using SVM, the True positive (TP) are 18283, False positive (FP) of 9; using XGB with default parameters we otained TP (16959), FP (1333), TN (1115), FN (593); using XGB with grid search we obtained TP (18273), FP (19), TN (1175), FN (533); using XGB with Bayesian optimization, we obtained TP (18281), FP (11), TN (1171), FN (537).

The Table 1 shows the performance of different machine learning algorithms for diabetes prediction using grid search and Bayesian optimization. The algorithms were evaluated using seven metrics: sensitivity, specificity, PPV, NPV, accuracy, F1-score, and MCC.

**Table 1:** Diabetes prediction using optimized machine learning algorithms with grid search and Bayesian optimization.

Classifiers	Sens.	Spec.	PPV	NPV	Accuracy	F1-score	MCC
Decision Tree	0.9704	1.0000	1.0000	0.6739	0.9721	0.9559	0.8087
SVM	0.9636	0.9912	0.9995	0.5954	0.9650	0.9455	0.7537
Naïve Bayes	0.9662	0.4555	0.9271	0.6528	0.9037	0.9143	0.4945
Logistic Regression	0.9636	0.8856	0.9928	0.5984	0.9591	0.9424	0.7085
KNN	0.9606	0.9706	0.9984	0.5615	0.9611	0.9406	0.7221
XGBoost	0.9726	0.9484	0.9964	0.6991	0**.9710**	**0.9574**	**0.8003**
XGB Grid search	0.9717	0.9841	0.9990	0.6879	**0.9724**	0.9572	0.8102
XGB Bayesian Optim. 1	**0.9716**	**0.9874**	**0.9992**	**0.6874**	**0.9726**	**0.9572**	**0.8114**
XGB Bayesian Optim. 2	**0.9715**	**0.9907**	**0.9994**	**0.6856**	**0.9726**	**0.9571**	**0.8118**

- Sensitivity: All models achieved high sensitivity (>96%), indicating they were good at correctly identifying true positive cases (people with diabetes).
- Specificity: Decision Tree had the highest specificity (1.00), meaning it made the fewest false positive predictions (classifying healthy people as diabetic). Naive Bayes had the lowest specificity (0.4555), suggesting it made many false positive predictions.
- PPV and NPV: XGBoost models had the highest PPV and NPV, meaning they were good at correctly classifying both true positive and true negative cases.
- Accuracy: All models achieved high accuracy (>95%), but XGBoost models had the highest accuracy (0.9726).
- F1-score: XGBoost models also had the highest F1-score, suggesting they were the best overall performers for this task.
- MCC: XGBoost models had the highest MCC, which is a more balanced measure of performance than accuracy.

The XGBoost models with grid search and Bayesian optimization appear to be the best performing algorithms for diabetes prediction in this dataset. However, it is important to note that these results may not generalize to other datasets, and further research is needed to confirm the generalizability of these findings.

The XGBoost variants generally perform well. All XGBoost models (including Grid Search and Bayesian Optimization variations) achieve high accuracy (above 97%) and F1-score (above 95%). This suggests they are effective at identifying both diabetic and non-diabetic cases. Decision Tree has high sensitivity but low Negative Predictive Value: While the Decision Tree has near-perfect sensitivity (97%), its NPV is only around 67%. This means it might miss some non-diabetic cases, flagging them as diabetic. Naïve Bayes has good sensitivity but poor Specificity: Similar to the Decision Tree, Naïve Bayes has high sensitivity (96%) but low Specificity (45%). It might be good at catching diabetes cases but also misidentifies many healthy individuals. Grid Search and Bayesian Optimization improve XGBoost performance: Both optimization techniques seem to slightly improve XGBoost’s performance on metrics like Specificity, MCC, and F1-score compared to the base XGBoost model. XGBoost with hyperparameter optimization appears to be the most effective model for this dataset based on a combination of high accuracy, sensitivity, specificity, and F1-score. However, the choice of the best model might depend on the specific priorities for your application. For example, if it’s crucial to avoid missing diabetic cases (even at the risk of misclassifying some healthy individuals), the Decision Tree might be a better choice despite its lower NPV.

Traditional machine learning algorithms, such as logistic regression and decision trees, provide foundational insights into diabetes prediction by identifying key influencing factors. While valuable for interpretability, their predictive capabilities may be limited. Conversely, ensemble methods like XGBoost excel at capturing complex patterns within data, often surpassing traditional models in accuracy. By combining these approaches, researchers can leverage the strengths of both worlds, enhancing diabetes prediction models with both interpretability and predictive power.

Bayesian optimization surpasses traditional hyperparameter tuning methods by employing a probabilistic model to efficiently explore the parameter space. This approach accelerates the discovery of optimal hyperparameters while reducing computational overhead. By intelligently balancing exploration and exploitation, Bayesian optimization effectively navigates the hyperparameter landscape. Its robustness to complex problem structures and ability to incorporate prior knowledge further solidify its advantage over grid and random search methods. The evaluation of various machine learning algorithms for diabetes prediction reveals several key points. Most models demonstrated strong ability to identify individuals with diabetes (high sensitivity), crucial for early detection. However, the capacity to correctly identify those without diabetes (specificity) varied significantly. XGBoost models, particularly those optimized through grid search and Bayesian optimization, consistently outperformed others across multiple metrics. A trade-off between sensitivity and specificity was observed in some models, emphasizing the need to balance these factors based on specific requirements.

XGBoost, a potent gradient boosting algorithm, excels in handling intricate datasets and delivering robust predictions, making it a favored tool for data scientists. However, its true potential is unleashed through meticulous hyperparameter optimization. While traditional methods like grid and random search are computationally demanding, Bayesian optimization offers a more efficient approach, intelligently exploring the hyperparameter space to maximize model performance. By combining XGBoost with Bayesian optimization, practitioners significantly enhance model accuracy, precision, recall, and generalization, ultimately creating superior machine learning models across diverse applications. XGBoost models, particularly those optimized through Bayesian optimization, consistently outperformed others across multiple metrics.

XGBoost model performance is significantly influenced by a subset of its hyperparameters. Key factors include tree depth, learning rate, number of trees, and regularization to prevent overfitting. Controlling the proportion of data and features used for each tree also impacts performance. While minor adjustments to hyperparameter ranges can yield incremental improvements, as demonstrated by XGBoost optimization 1 and 2, the proposed ensemble XGBoost model with Bayesian optimization offers a more robust approach to enhancing diabetes prediction compared to traditional machine learning algorithms.

## 5. Conclusions

Diabetes, a chronic condition affecting millions globally, necessitates early detection and management to prevent severe complications. Accurately predicting diabetes onset or progression remains a significant challenge due to the complexity and imbalance of available data. Traditional predictive models, often relying on fixed parameters, have limitations in capturing the intricate patterns associated with diabetes. A promising approach involves optimizing an XGBoost model using Bayesian optimization. By leveraging lifestyle and clinical data, this method effectively identifies key factors influencing diabetes risk. The resulting model offers improved accuracy in predicting diabetes, enabling more precise patient management and tailored prevention strategies.

While a recent study using this optimized XGBoost approach showed a modest initial improvement compared to traditional methods, the potential for further refinement is significant. This approach has the potential to revolutionize diabetes prevention and treatment. By providing more accurate predictions, it holds promise for a brighter future for individuals at risk of developing this chronic condition.

### 5.1. Limitations and future directions

The presented study offers a promising approach to diabetes prediction by employing XGBoost with Bayesian optimization. However, certain limitations and avenues for future research are evident.

### 5.2. Study limitations

- **Modest Improvement:** While the study demonstrated progress over traditional methods, the magnitude of improvement was relatively small. Further research is necessary to establish more substantial clinical benefits.
- **Data Constraints:** The study’s reliance on data quality and quantity underscores the importance of addressing potential biases and limitations in data sources.
- **Generalizability Concerns:** The model’s applicability to diverse populations and healthcare settings remains uncertain due to potential variations in patient characteristics and data availability.

### 5.3. Future research directions

To build upon the study’s foundation, future research should focus on:

- **Optimizing Model Performance:** Refining hyperparameters and exploring alternative XGBoost configurations to enhance predictive accuracy.
- **Expanding Data Sources:** Incorporating genetic, environmental, and longitudinal data to enrich model capabilities.
- **Validating Model Generalizability:** Assessing model performance across diverse populations to ensure reliability and applicability.
- **Clinical Integration:** Developing user-friendly tools to seamlessly integrate the model into healthcare workflows.
- **Longitudinal Assessment:** Tracking model performance over time to evaluate its ability to predict disease progression and treatment response.
- **Ethical Framework:** Establishing robust ethical guidelines to safeguard patient privacy and promote responsible AI practices.

By addressing these limitations and pursuing these research directions, the potential of XGBoost and Bayesian optimization in diabetes prediction can be fully realized, leading to improved patient outcomes.
